# Treatment planning comparison of volumetric modulated arc therapy employing a dual-layer stacked multi-leaf collimator and helical tomotherapy for cervix uteri

**DOI:** 10.1186/s13014-020-1473-z

**Published:** 2020-01-30

**Authors:** S. Panda, J. Swamidas, S. Chopra, A. Mangaj, A. Fogliata, P. Kupelian, J. P. Agarwal, L. Cozzi

**Affiliations:** 1 Department of Radiation Oncology, Advanced Centre for Treatment Research and Education in Cancer, Tata Memorial Centre, Homi Bhabha National Institute, Kharghar, Navi Mumbai, Maharashtra India; 2 Department of Radiation Oncology and Medical Physics, Tata Memorial Hospital, Tata Memorial Centre, Homi Bhabha National Institute, Parel, Mumbai, Maharashtra India; 3 Humanitas Research Hospital, Radiotherapy and Cancer Center Radiosurgery Dept, Via Manzoni 56, 20089 Milan-Rozzano, Italy; 4 Varian Medical Systems, Palo Alto, CA USA; 5 Radiation Oncology Dept., University of California, Los Angeles, USA; 6 Dept. of Biomedical Sciences, Humanitas University, Milan-Rozzano, Italy

**Keywords:** Dual-layer MLC, Halcyon, Helical Tomotherapy, Cervix uteri, VMAT, RapidArc

## Abstract

**Purpose:**

To ascertain the dosimetric performance of a new delivery system (the Halcyon system, H) equipped with dual-layer stacked multi-leaf collimator (MLC) for risk-adapted targets in cervix uteri cancer patients compared to another ring-based system in clinical operation (Helical Tomotherapy, HT).

**Methods:**

Twenty patients were retrospectively included in a treatment planning study (10 with positive lymph nodes and 10 without). The dose prescription (45Gy to the primary tumour volume and a simultaneously integrated boost up to 55Gy for the positive patients) and the clinical planning objectives were defined consistently as recommended by an ongoing multicentric clinical trial. Halcyon plans were optimised for the volumetric modulated arc therapy. The plan comparison was performed employing the quantitative analysis of the dose-volume histograms.

**Results:**

The coverage of the primary and nodal target volumes was comparable for both techniques and both subsets of patients. The primary planning target volume (PTV) receiving at least 95% of the prescription isodose ranged from 97.2 ± 1.1% (node-negative) to 99.1 ± 1.2% (node-positive) for H and from 96.5 ± 1.9% (node-negative) to 98.3 ± 0.9% (node-positive) for HT. The uncertainty is expressed at one standard deviation from the cohort of patient per each group. For the nodal clinical target volumes, the dose received by 98% of the planning target volume ranged 55.5 ± 0.1 to 56.0 ± 0.8Gy for H and HT, respectively. The only significant and potentially relevant differences were observed for the bowels. In this case, V_40Gy_ resulted 226.3 ± 35.9 and 186.9 ± 115.9 cm^3^ for the node-positive and node-negative patients respectively for Halcyon. The corresponding findings for HT were: 258.9 ± 60.5 and 224.9 ± 102.2 cm^3^. On the contrary, V_15Gy_ resulted 1279.7 ± 296.5 and 1557.2 ± 359.9 cm^3^ for HT and H respectively for node-positive and 1010.8 ± 320.9 versus 1203.8 ± 332.8 cm^3^ for node-negative.

**Conclusion:**

This retrospective treatment planning study, based on the dose constraints derived from the Embrace II study protocol, suggested the essential equivalence between Halcyon based and Helical Tomotherapy based plans for the intensity-modulated rotational treatment of cervix uteri cancer. Different levels of sparing were observed for the bowels with H better protecting in the high-dose region and HT in the mid-low dose regions. The clinical impact of these differences should be further addressed.

## Introduction

Primary chemoradiation and brachytherapy is the standard of care for patients with locally advanced cervical cancer. External beam radiation therapy (EBRT), brachytherapy, and concomitant chemotherapy are the integral parts of this treatment. However, these are associated with clinically significant acute and late toxicity. While most of the late sequelae in organs at risk (rectum, sigmoid and bladder) may be attributed to brachytherapy, a significant proportion of gastrointestinal acute and late sequelae are a result of both low and high doses received by the bowel. As the newer treatment algorithms envisage brachytherapy and nodal dose escalation, it’s imperative that doses to organs at risk that potentially receive a contribution from both external radiation and brachytherapy are minimised to the lowest possible level. Also, in patients receiving extended field radiation due to para-aortic disease, higher incidence of gastrointestinal toxicity may be expected, and highly conformal radiation fields may help in reducing bowel dose. The use of Image-guided Intensity Modulated Radiation Therapy (IG-IMRT) is associated with reduced doses to the small bowel and may be associated with a reduction in patient-reported symptoms and physician-reported toxicity outcomes [1–5]. Similarly, IG-IMRT for EBRT is associated with a reduced incidence of gastrointestinal toxicity.

Different variants of intensity-modulated radiotherapy (IMRT) techniques exist and, among these, the role of volumetric modulated arc therapy (VMAT) has been compared to fixed beam IMRT [6] confirming the potential of other rotational therapy techniques as Helical Tomotherapy (HT) [7–9].

After its clinical introduction in 2017, the performance of the new radiotherapy delivery platform Halcyon (H, Varian Medical Systems, Palo Alto, USA) was investigated for a variety of different treatment indications [10–20]. In general, the potential performance equivalence between H and other c-arm delivery systems was proven for head and neck, breast, prostate [13] and brain tumours [15]. Some studies focused on cervix uteri cancer planning. With early versions of the Halcyon environment, Anamalayil [16], Brady [17] and Mihailidis [18] showed that: i) a good agreement was achievable between plans optimised for H or for other conventional platforms for both IMRT and VMAT and that ii) the use of multiple isocenters (with auto-feathering of overlapping fields/arcs) could overcome the field size limitations of H. More recently, using the second generation release of the Halcyon system, Kim [19] reported about the clinical use of enhanced planning capabilities of H for the treatment of long targets using dual-isocenters. Li [20] compared the performance of Halcyon versus a conventional c-arm linac for IMRT.

The primary aim of the present study was to investigate the performance of Halcyon and Helical Tomotherapy for pelvic radiation for node-negative and node-positive patients according to the planning strategy of an ongoing multicentric international clinical trial, the Embrace II study [21], i.e. in a complex clinical strategy for gynaecological cancer. The protocol involves complex planning algorithm with specific dose constraints for various subvolumes for pelvic radiation. Furthermore, specific aims are also enlisted for nodal dose escalation while maintaining organ at risk doses.

The quality of the plans was compared for two rotational platforms. To further appraise the merit and potential issues of the new system, the Halcyon based plans were compared against the corresponding ones optimised for Helical Tomotherapy.

## Materials and methods

### Patient selection, target definition and dose prescription

A retrospective treatment planning study was performed on a cohort of 20 patients; 10 cases were selected with positive lymph nodes (N+) and 10 without positive nodes (N-) from the institutional trial database. Target delineation was performed according to the definitions and guidelines of the ongoing international clinical trial for gynaecological IG-IMRT [21]. In summary, target delineation was performed after fusing contrast-enhanced computed tomography (CT) images with a resolution of 1.2 × 1.2 × 2.5 mm^3^ and T2 weighted magnetic resonance images (MRI, resolution of 5x5x3 mm^3^) registered to those images. The gross tumour volume (GTV) was delineated as the intermediate signal intensity region in the cervix and vagina on T2 MRI. The clinical target volumes (CTV) were delineated as follows: i) the high-risk CTV (CTV_HR, defined as the primary GTV and any remaining cervix not infiltrated by the tumour) and the low-risk CTV (CTV_LR) for the primary GTV including the parametria, the uterus and a margin of about 5 mm anterior and posterior towards bladder and rectum; ii) the elective CTV (CTV_E) for the elective nodal volume (including the nodal regions according to the risk stratification) and iii) the nodal CTV (CTV_N, with a further indication of the number of lymph-nodes (e.g. CTV_N1)) for the lymph nodes (with a margin of 3 mm from the nodal GTV). The internal target volume (ITV) of the primary tumour (ITV45) was defined as the union of the CTV_E and the ITV from the CTV_LR (obtained adding a 10-mm margin along the anterior-posterior and superior-inferior axes and 5 mm along the lateral axis), excluding muscles and bony boundaries in the pelvis. The planning target volumes (PTV45 and PTV_N respectively for the primary tumour and the nodal volumes) were generated with an isotropic margin of 5 mm from the corresponding ITVs or CTVs.

The dose prescription was set to 45Gy in 25 fractions of 1.8Gy for PTV45. The nodal boost dose (for the N+ subset) was optimised according to the simultaneous integrated boost (SIB) technique with a total dose of 55Gy/25fractions (2.2Gy per fraction). Dose optimisation and final computation were performed on contrast-free CT scans registered to the images used for segmentation.

The dose coverage to PTV45 was aimed to achieve that 95% of the volume is covered by the 95% isodose (V_95%_ > 95). A stricter coverage was requested for the ITV45: D_99%_ > 95. For the planning target volumes of the pathological nodes, the required dose coverage was: D_98%_ > 49.5Gy (i.e. 90% of the prescription) while for the nodal clinical target volumes it was: D_98%_ > 55Gy (100%). The near-to-maximum dose objective was defined as D_1%_ < 107%.

The organs at risk (OAR) considered for the study were the bowel bag (defined as the outer contour of the loops including the mesenterium), the sigmoid, the bladder, the rectum, the spinal cord, the femoral heads and the kidneys. For each of these OARs, the specific dose-volume constraints were set for the near-to-maximum doses and for the organ-specific additional parameters. For the bladder, the rectum and the bowels, the volume objectives were enforced as soft constraints (due to the interpatient variability in the organs to target contouring), aiming to be achieved in about 70–80% of the cases. A detailed list of the constraints to the OARs is reported in the results together with the observed experimental findings.

### The treatment planning techniques and features

Two sets of plans were designed and optimised for each patient in a full double-blind manner. Data analysis and comparison was performed only at the end of the planning phase, not allowing for further plan improvements. All plans were specifically designed and optimised for this in-silico treatment planning investigation.

*Halcyon plans:* The Halcyon is a ring-based delivery system [13, 22–24]. It consists of a linear accelerator capable of producing a 6MV flattening filter-free (FFF) photon beam delivering (when calibrated to deliver 1.0Gy per 100 monitor units (MU) at the reference conditions of source-surface distance (SSD) 100 cm at d_max_ depth of 1.3 cm) a maximum dose rate of 800MU/min. The linac rotates around the mounting ring with a velocity up to 4 rotations per minute in both imaging and delivery mode. The main features of Halcyon relevant for planning are: i) absence of the jaws in the X and Y directions; ii) a dual-layer stacked-staggered multi-leaf collimator; each layer is “shifted” 5 mm with respect to the other producing an effective shaping capability of 5 mm at isocenter. The design specifications of the multi-leaf collimator (MLC) stated a 0.01 transmission and leakage; iii) maximum field size for a single isocenter treatment of 28x28cm^2^ and 28.0 cm of overtravel of the MLC leaves with full inter-digitation; iv) possibility to automatically generate two isocenter plans for longer targets (up to 36.0 cm in Halcyon version 2) with auto-feathering of overlapping fields/arcs.

All the plans were designed and optimised using the Eclipse treatment planning system (Varian Medical Systems, Palo Alto, USA) version 15.6. For each patient, VMAT plans were generated using a class solution consisting of 3 full arcs with collimator angles set to 10°, 350° and 90°. A single isocenter was used for all N- cases and for 8/10 N+ cases. In the remaining two cases, the target volume resulted longer than 28 cm and therefore the plans were optimised for two isocentres (with the same arcs arrangement) displaced of 8 cm in the z-direction. The optimisation (and the delivery) is fully automated in Eclipse; field feathering is applied to accurately match the dose distribution in the overlapping region.

The VMAT optimisation in Eclipse was performed without applying constraints to the MLC complexity, i.e. leaving the system the freedom to adapt the “modulation intensity” case by case which is the planning default. For all plans, the dose distributions were computed using the Anisotropic Analytical Algorithm (AAA) [25] with a resolution of 2.5 mm.

*Helical Tomotherapy Plans*: HT machine consists of a 6MV FFF beam with binary MLC, capable of a minimum leaf opening time of 20 ms. The jaw widths are of size 1 × 40, 2.5 × 40 and 5x40cm^2^ at the Source to Axis distance of 85.0 cm. The dose rate was 850 cGy/min. For the current study, the field size was set to 5x40cm (with static jaws) and a pitch of 0.3 was used for all patients. The modulation factor was set to 3 for all plans. In HT, the delivery is helical in nature that enables long treatment lengths of up to 160 cm, without any junction. Imaging is possible with a megavoltage fan-beam CT, for a maximum length of 160 cm. The detailed machine characteristics can be found elsewhere [26–29]. All the plans were designed and optimised on the Hi-Art treatment planning system version 5.1.4 (Accuray, Sunnyvale, USA) with a resolution of 5 mm in the x-y plane and 2.5 mm in z.

*Quantitative assessment:* The analysis of the dose distributions aimed to quantify a variety of relevant metrics derived from the dose-volume histograms (DVH) for the various target volumes and OARs. For each structure, the mean dose and selected V_x_ and D_x_ parameters were extracted. V_x_ represents the volume receiving at least an x level of dose and D_x_ the dose received by an x fraction of volume. The metrics reported are relative to the dose-volume objectives suggested by the ongoing international clinical trial. For the planning target volumes, also the homogeneity index (Homog = (D_5%_-D_95%_)/D_mean_) was scored to measure the variance of the dose inside the volumes supposed to be homogeneously irradiated. Homogeneity was not part of the planning objectives set, but it was reported for consistency with literature standards. The conformity index (CI) was defined as the ratio of the volume receiving at least 36Gy or 43Gy to the volume of PTV45 (CI_36Gy_ and CI_43Gy_ respectively) and in terms of the Paddick conformity index (CI_Paddick_) [30] computed for the 95% isodose, i.e. 42.74 Gy. The Paddick index was computed also for the sum of the SIB boost volumes (PTV_N1 and PTV_N2) for the 52.5Gy dose level (95% of the boost dose prescription) and reported as CI_Paddick_SIB_. To complete the analysis, also the average DVH were computed, for each structure and each cohort with a dose binning resolution of 0.02Gy. All dose-volume metrics were derived from the systems the plans were optimised in. In-house tools were used to generate the average DVH for the images. All dose distributions were imported in the Eclipse system for the graphical comparison. For both techniques the estimated treatment time (excluding imaging) was derived from the plans and reported as a further comparative factor.

The assessment of the potential statistical significance of the differences among the groups of plans was performed using non-parametric tests (Wilcoxon test for paired samples).

## Results

**Fig. 1:**
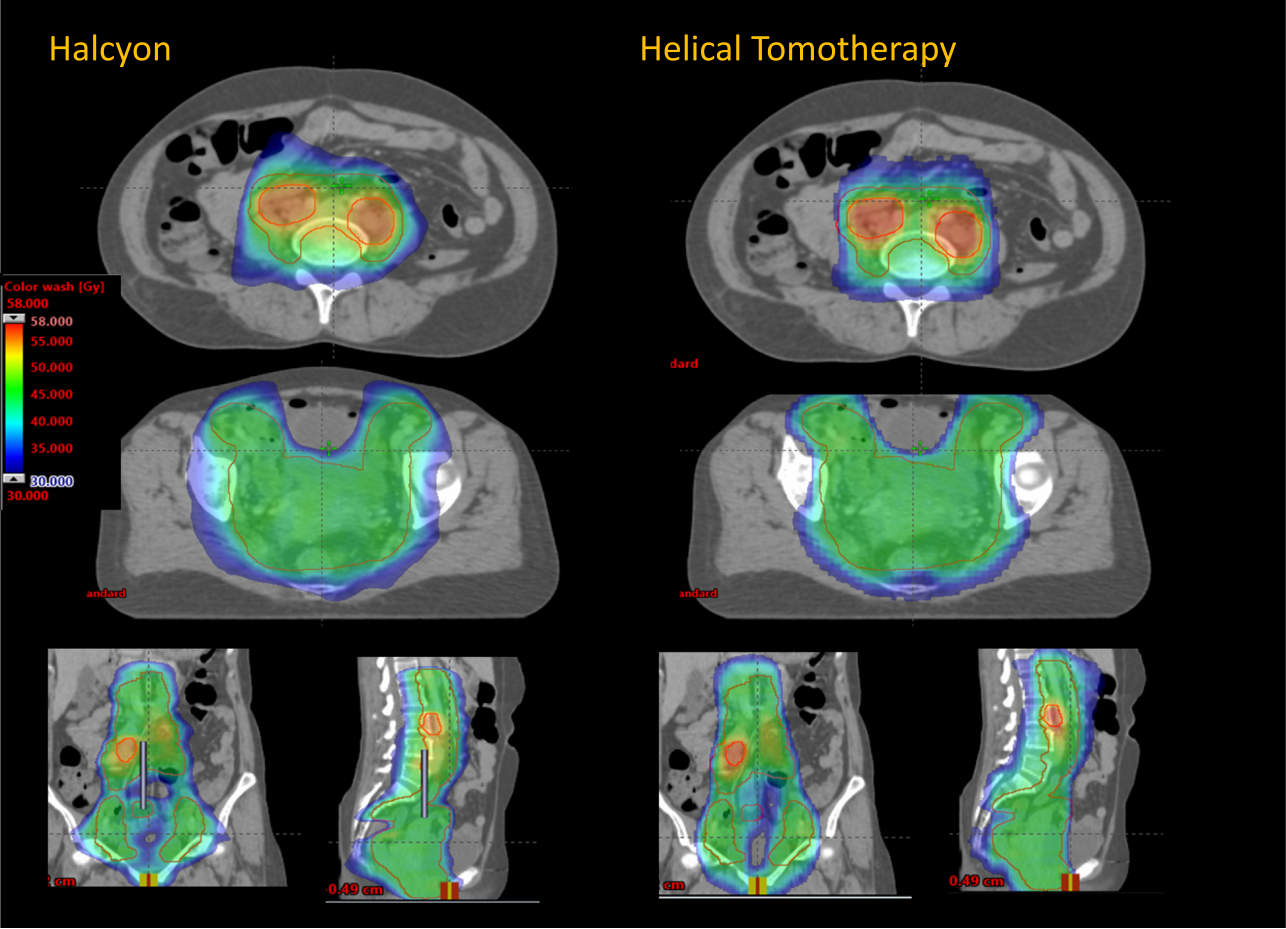
Isodose distributions in colour wash (scale between 35 and 48Gy) for one example of node-positive patient in two axial, coronal and sagittal planes

**Fig. 2:**
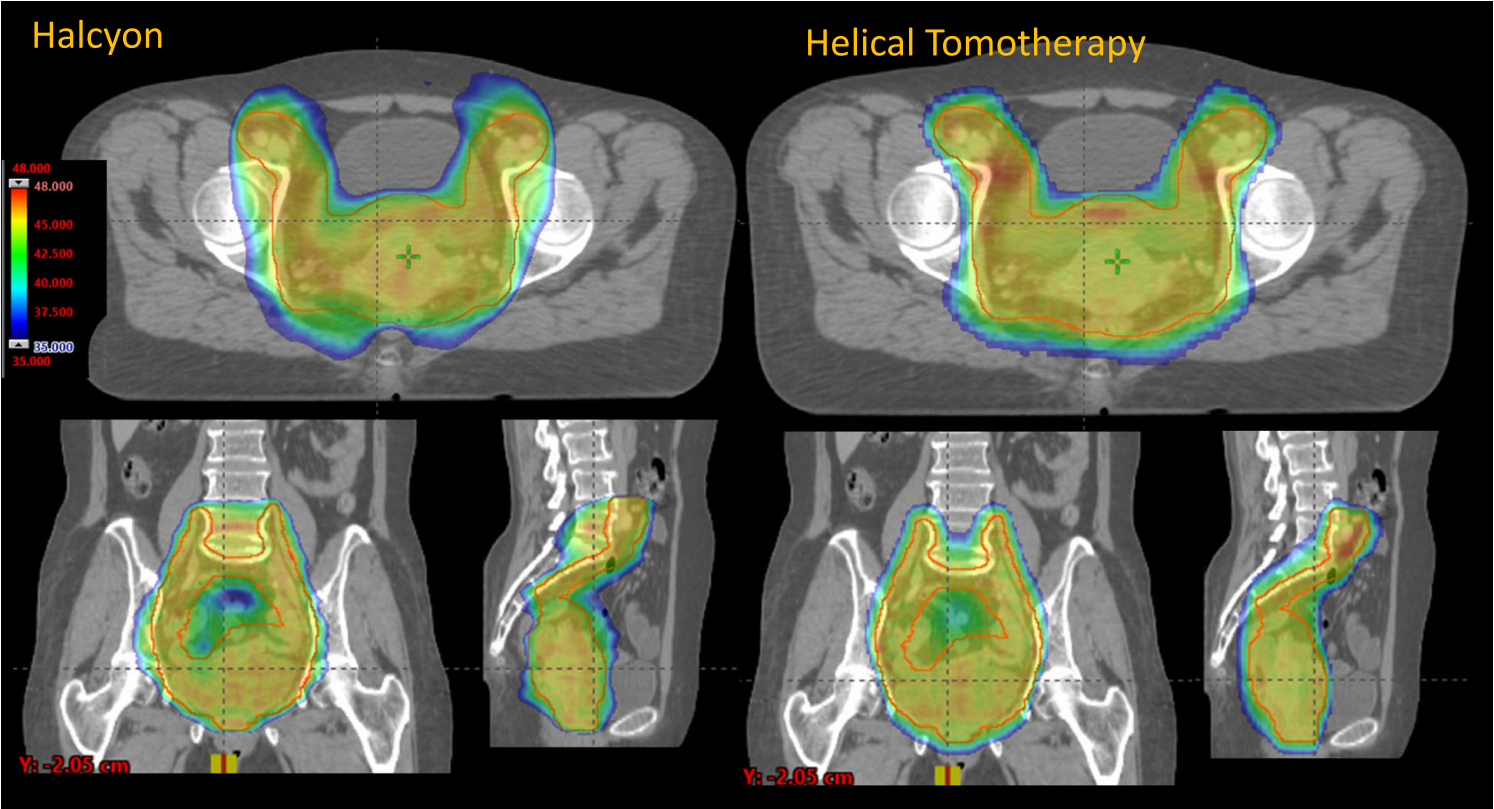
Isodose distribution in colour wash (scale between 35 and 48Gy) for one example of node-negative patients in an axial, coronal and sagittal planes

Figures 1 and 2 present the isodose distributions in colour-wash for two example patients from the N+ and N- cohorts. The qualitative assessment demonstrates very similar degrees of conformality and sparing of organs at risk for both H and HT plans.

**Fig. 3:**
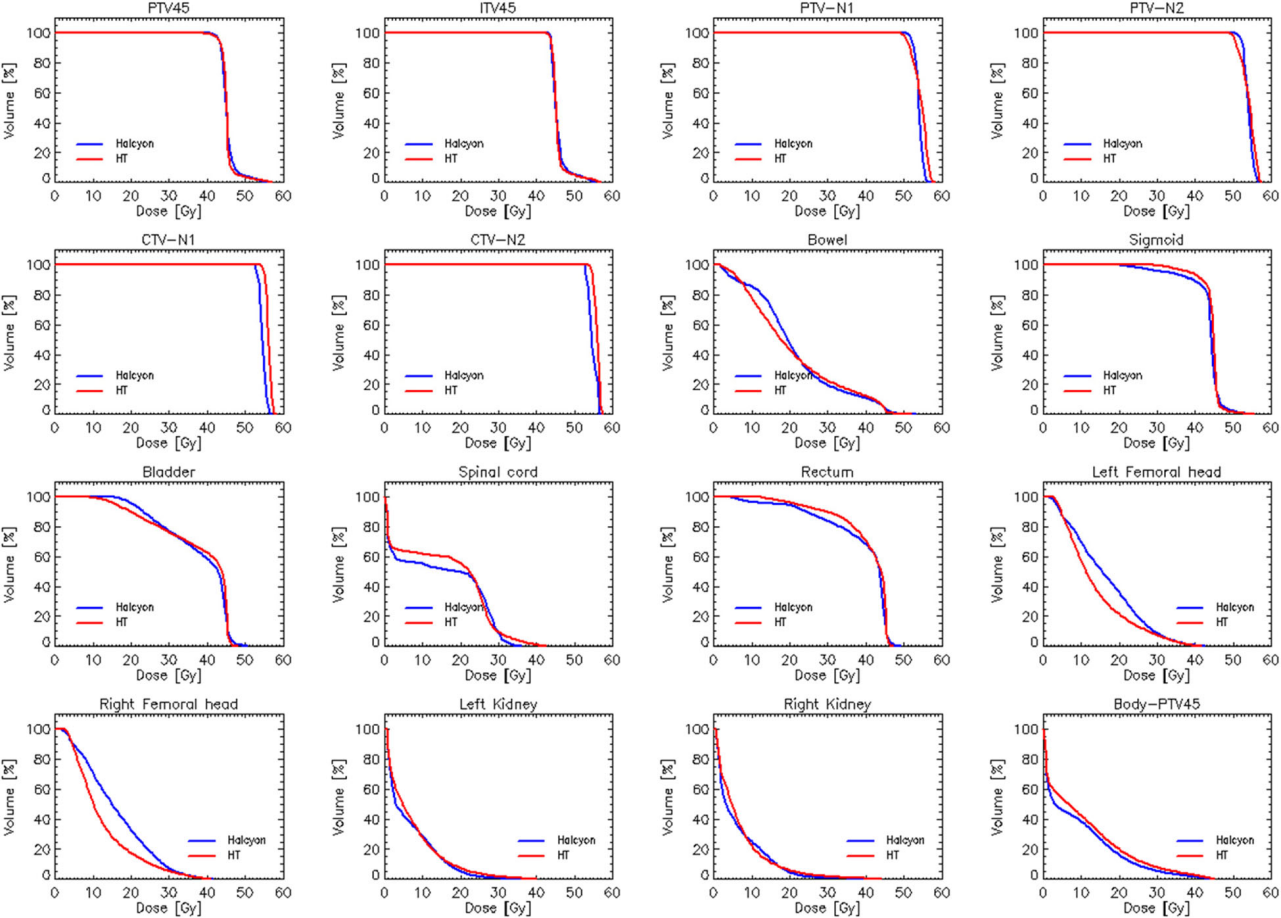
Average dose-volume histograms for the cohort of node-positive patients. Data are shown for all the target volumes and the organs at risk

**Fig. 4:**
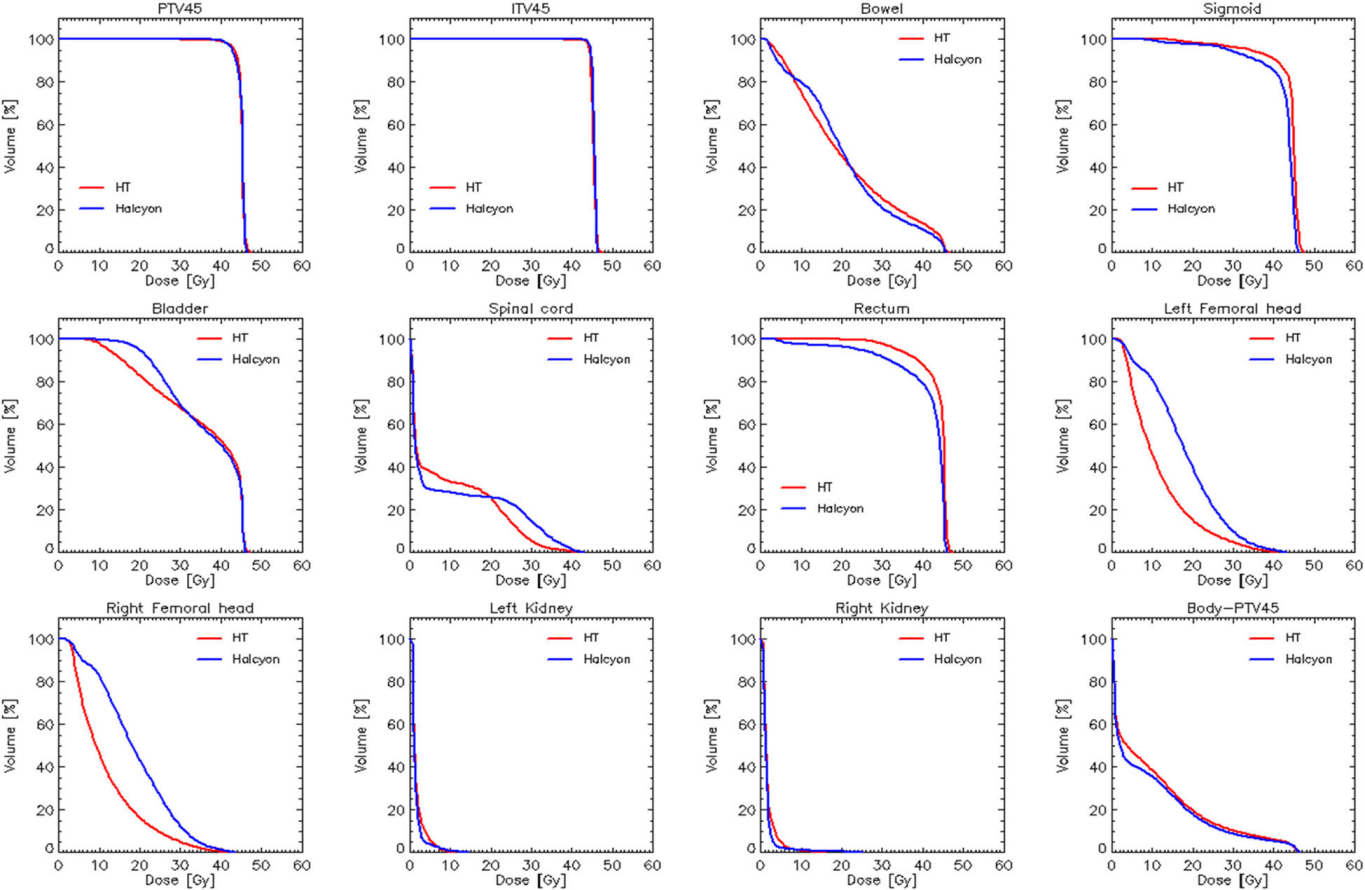
Average dose volume histograms for the cohort of node-negative patients. Data are shown for all the target volumes and the organs at risk

Figures 3 and 4 show the average dose-volume histograms computed for the two subgroups of patients. Data are shown for the various target volumes and the organs at risk. The quantitative analysis of the DVHs is summarised in Table 1 (for the target volumes) and Tables 2-3 for the OARs.

**Table 1:** Summary of the dose volume histogram analysis for the target volumes. Data are presented for the node positive and node negative patients

Parameter	Objective	Halcyon (H)	Helical Tomotherapy (HT)	Difference (H-HT)	p
Node Positive					
PTV45					
V_95%_ [%]	≥95	99.1 ± 1.2	98.3 ± 0.9	0.8 ± 0.9	0.02
D_1%_ [%]	≤130^b^	122.9 ± 1.7	123.2 ± 3.8	−0.3 ± 2.8	0.73
Homog [%]	^a^	2.8 ± 3.1	3.3 ± 2.1	−0.5 ± 2.6	0.002
ITV45					
D_99%_ [%]	≥95	100.1 ± 1.2	97.6 ± 1.3	2.5 ± 1.1	< 0.001
D_50%_ [Gy]	45.0	45.0 ± 0.4	45.8 ± 0.5	−0.7 ± 0.6	0.002
PTV_N1					
D_1%_ [Gy]	≤58.9	56.2 ± 0.2	58.0 ± 0.6	−1.8 ± 0.6	< 0.01
D_98%_ [Gy]	≥49.5	53.8 ± 0.3	51.2 ± 1.2	2.6 ± 1.1	< 0.01
Homog [%]	^a^	3.8 ± 2.7	8.9 ± 3.5	−5.1 ± 3.3	< 0.001
PTV_N2					
D_1%_ [Gy]	≤58.9	56.3 ± 0.2	58.1 ± 0.7	−1.2 ± 1.0	< 0.001
D_98%_ [Gy]	≥49.5	53.6 ± 0.3	51.0 ± 1.2	1.8 ± 1.5	< 0.001
Homog [%]	^a^	3.9 ± 3.1	9.1 ± 4.0	5.2 ± 4.0	< 0.001
CTV_N1					
D_98%_ [Gy]	≥55.0	55.5 ± 0.1	56.0 ± 0.8	−0.5 ± 0.9	0.10
D_50%_ [Gy]	55.0	54.5 ± 0.9	57.1 ± 0.5	− 2.5 ± 1.2	< 0.001
CTV_N2					
D_98%_ [Gy]	≥55.0	55.5 ± 0.2	55.5 ± 0.4	−0.1 ± 0.3	0.63
D_50%_ [Gy]	55.0	54.4 ± 1.1	57.1 ± 0.5	−2.7 ± 1.2	< 0.001
Node Negative					
PTV45					
V_95%_ [%]	≥95	97.2 ± 1.1	96.5 ± 1.9	0.7 ± 1.9	0.27
D_1%_ [%]	≤107	104.2 ± 0.9	103.1 ± 0.7	0.1 ± 0.1	0.003
Homog [%]	^a^	4.5 ± 1.5	3.2 ± 2.1	1.3 ± 0.9	0.007
ITV45					
D_99%_ [%]	≥95	100.0 ± 0.8	96.5 ± 1.5	3.3	< 0.001
D_50%_ [Gy]	45.0	45.4 ± 0.1	45.1 ± 0.2	0.3 ± 2	0.23

^a^: not included as optimisation constraint

^b^: due to the simultaneous irradiation of the boost volumes. V_x%_: volume receiving at least x% of the dose. D_x%_: dose received by at least x% of the volume. Homog = (D5-D_95_)/D_mean_

**Table 2:** Summary of the dose volume histogram analysis for the organs at risk for the node positive patients

Parameter	Objective	Halcyon (H)	Helical Tomotherapy (HT)	Difference (H-HT)	p
Bowel					
Mean [Gy]	Minimise	21.2 ± 3.1	21.1 ± 2.8	0.1 ± 0.9	0.82
D_1%_ [Gy]	≤57.5	48.8 ± 1.7	47.4 ± 1.3	1.4 ± 0.7	< 0.001
V_40Gy_ [cm^3^]	≤250 ^a^	226.3 ± 35.9	258.9 ± 60.5	−32.6 ± 34.3	0.01
V_30Gy_ [cm^3^]	≤500 ^a^	413.4 ± 58.9	472.7 ± 90.0	−59.4 ± 53.5	0.01
V_15Gy_ [cm^3^]	≤700 ^b^	1557.2 ± 359.9	1279.7 ± 296.5	277.4 ± 188.6	0.01
Sigmoid					
D_1%_ [Gy]	≤57.5	50.5 ± 3.7	49.4 ± 3.8	1.1 ± 2.1	0.11
Bladder					
D_1%_ [Gy]	≤57.5	48.8 ± 2.2	47.0 ± 1.1	1.8 ± 1.8	0.01
V_40Gy_ [%]	≤75 ^a^	60.0 ± 10.8	63.6 ± 16.6	−3.6 ± 7.5	0.16
V_30Gy_ [%]	≤85 ^a^	76.9 ± 7.1	77.0 ± 12.5	−0.1 ± 9.2	0.96
Rectum					
D_1%_ [Gy]	≤57.5	48.1 ± 1.3	46.9 ± 0.7	1.2 ± 1.8	0.05
V_40Gy_ [%]	≤85 ^a^	66.6 ± 20.5	72.8 ± 21.7	−6.2 ± 11.2	0.11
V_30Gy_ [%]	≤95 ^a^	81.9 ± 12.7	89.7 ± 14.7	−7.8 ± 12.2	0.07
Spinal Cord					
D_1%_ [Gy]	≤48.0	25.1 ± 17.9	25.6 ± 16.5	−2.4 ± 4.4	0.12
Left Fem. Head					
D_1%_ [Gy]	≤50.0	38.1 ± 3.6	39.4 ± 1.6	−1.3 ± 3.2	0.24
Mean [Gy]	^b^	16.4 ± 2.7	13.8 ± 2.5	2.6 ± 3.1	0.001
Right Fem. Head					
D_1%_ [Gy]	≤50.0	36.3 ± 4.6	41.7 ± 2.9	−5.4 ± 2.5	< 0.001
Mean [Gy]	^b^	16.1 ± 2.3	12.6 ± 1.3	3.5 ± 1.9	< 0.001
Left Kidney					
Mean [Gy]	≤15	6.9 ± 5.0	7.5 ± 5.1	−0.6 ± 1.5	0.22
Right Kidney					
Mean [Gy]	≤	6.6 ± 4.8	7.1 ± 4.2	− 0.5 ± 2.1	0.52
Body					
D_1%_ [Gy]	≤58.9	53.2 ± 1.4	49.9 ± 3.7	3.3 ± 4.1	< 0.001
CI_36Gy_	< 1.6 ^b^	1.6 ± 0.2	2.0 ± 0.1	−0.4 ± 0.2	< 0.001
CI_43Gy_	< 1.1 ^b^	1.1 ± 0.1	1.3 ± 0.1	−0.2 ± 0.1	< 0.001
CI_Paddick_	^b^	0.87 ± 0.04	0.90 ± 0.07	−0.03 ± 0.08	0.01
CI_Paddick_SIB_	^b^	0.71 ± 0.08	0.78 ± 0.15	−0.07 ± 0.10	0.05

V_x%_: volume receiving at least x% of the dose. D_x%_: dose received by at least x% of the volume

^a^: soft constraints, expected to be fulfilled in 70–80% of the patients according to EMBRACE II guidelines

^b^: not included as optimisation constraint

**Table 3:** Summary of the dose volume histogram analysis for the organs at risk for the node negative patients

Parameter	Objective	Halcyon (H)	Helical Tomotherapy (HT)	Difference (H-HT)	p
Bowel					
Mean [Gy]	Minimise	20.5 ± 4.0	21.0 ± 3.0	−0.6 ± 1.7	0.7
V_40Gy_ [cm^3^]	≤100 ^a^	186.9 ± 115.9	224.9 ± 102.2	−38.1 ± 22.2	< 0.001
V_30Gy_ [cm^3^]	≤350 ^a^	360.9 ± 195.0	422.9 ± 134.2	−62.1 ± 72.1	0.02
V_15Gy_ [cm^3^]	≤600 ^b^	1203.8 ± 332.9	1010.8 ± 320.9	192.9 ± 160.4	0.4
Sigmoid					
D_1%_ [Gy]	≤47.3	46.1 ± 0.5	46.1 ± 0.8	−0.08 ± 0.89	0.78
Bladder					
D_1%_ [Gy]	≤47.3	46.6 ± 0.2	46.0 ± 0.3	0.58 ± 0.28	< 0.001
V_40Gy_ [%]	≤75 ^a^	50.4 ± 7.0	52.4 ± 8.3	−2.1 ± 6.6	0.35
V_30Gy_ [%]	≤85 ^a^	69.6 ± 5.8	67.6 ± 7.2	2.0 ± 8.6	0.47
Rectum					
D_1%_ [Gy]	≤47.3	46.2 ± 0.4	46.1 ± 0.6	0.10 ± 0.89	0.72
V_40Gy_ [%]	≤85 ^a^	80.8 ± 10.8	88.1 ± 12.3	−7.2 ± 11.6	0.08
V_30Gy_ [%]	≤95 ^a^	92.4 ± 6.4	97.4 ± 3.9	−5.0 ± 6.1	0.03
Left Fem. Head					
D_1%_ [Gy]	≤50.0	38.7 ± 6.9	32.6 ± 11.7	6.1 ± 12.7	0.17
Mean [Gy]	^b^	18.1 ± 2.7	11.7 ± 0.9	6.3 ± 2.2	< 0.001
Right Fem. Head					
D_1%_ [Gy]	≤50.0	38.5 ± 4.4	36.5 ± 3.6	1.9 ± 4.4	0.20
Mean [Gy]	^b^	18.8 ± 3..3	11.7 ± 0.9	6.9 ± 2.7	< 0.001
Left Kidney					
Mean [Gy]	≤15	1.5 ± 0.7	1.5 ± 0.8	−0.01 ± 0.32	0.97
Right Kidney					
Mean [Gy]	≤15	1.5 ± 1.0	1.6 ± 0.9	−0.11 ± 3.7	0.37
Body					
D_1%_ [Gy]	≤47.3	41.9 ± 1.1	42.5±	0.85 ± 0.59	0.001
CI_36Gy_	< 1.6 ^b^	1.56 ± 0.08	1.82 ± 0.19	−0.26 ± 0.16	0.19
CI_43Gy_	< 1.1 ^b^	1.08 ± 0.05	1.16 ± 0.09	−0.08 ± 0.08	0.09
CI_Paddick_	^b^	0.91 ± 0.03	0.86 ± 0.04	0.05 ± 0.03	0.03

V_x%_: volume receiving at least x% of the dose. D_x%_: dose received by at least x% of the volume. CI = conformity index

^a^: soft constraints, expected to be fulfilled in 70–80% of the patients according to EMBRACE II guidelines

^b^: not included as optimisation constraint

Starting from the N+ cases, the required dose coverage measured by V_95%_ for PTV45, D_98%_ for CTV_N and PTV_N and D_99%_ (near-to-minimum dose) for ITV45, was achieved for both H and HT plans. Data shows a systematic, although modest, improvement for H over HT as measured by the positive difference between H and HT results. The results for H demonstrated also a modest reduction (smaller than 1Gy for PTV45 and than 2Gy for the PTV_N) of the near-to-maximum doses, reported by D_1%_. The findings from coverage and maximum dose reflected in the improved homogeneity of H versus HT; to note that homogeneity was not part of the optimisation criteria. The median dose to the ITV45 resulted in average coherent with the aimed 45Gy with a modest but significant over-dosage observed in the HT data (0.7Gy higher than the H plans). The median dose for the nodal CTV (CTV_N1 and CTV_N2) resulted on average slightly inferior to the objective of 55Gy for H and about 2Gy higher for HT. In summary, the use of the SIB technique for the N+ cases did not impact in any significant manner on the quality of the plans in the comparison between the two delivery systems.

Concerning the simpler case of the N-, the PTV45 or ITV45 coverage (expressed by V_95%_ and D_99%_ respectively) was met by both techniques with H data improving the HT findings of 0.7% (not significant) or 3.3% respectively. In this case, the homogeneity of the H plans resulted about 1% worse than HT, in an opposite manner to what reported for the N+ cases. The median dose to the ITV45 resulted consistent with the constraint of 45Gy for both H and HT without significant differences.

Although most of the differences between H and HT plans resulted statistically significant, their modest absolute value might not impact at the clinical level.

Concerning the OARs, the results are more variable. For the sigmoid, the spinal cord, the femoral heads and the kidneys both delivery systems mostly met the planning objectives. For the femoral heads, although not subject to any constraints in the optimisation, the HT plans better spared the entire structures resulting in significantly lower mean doses. In most of the cases, no statistical significance was measured in the comparison of the groups. The optimisation of the dose distribution for the bowels, the bladder and the rectum resulted more challenging.

For the bowels, the EMBRACE II required as a hard constraint the maximum dose to be lower than 47.3 or 57.5 Gy for the N- and N+ plans respectively. Both delivery systems achieved this request for all patients in terms of the near-to-maximum parameter.

Soft objectives were enforced to spare the volume receiving 30 or 40 Gy below 500 and 250 cm^3^ for the N+ patients (350 and 100 cm^3^ respectively for the N-). For N+ patients, H resulted in a significant additional sparing of in average 60 and 33 cm^3^ for N+ and 62 and 38 cm^3^ for the N- patients. HT plans presented a lower dose spillage (V_15Gy_) which, although a parameter not included in the optimisation constraints, is a desirable result: V_15Gy_ resulted 1279.7 ± 296.5 and 1557.2 ± 359.9 cm^3^ for HT and H respectively for node-positive and 1010.8 ± 320.9 versus 1203.8 ± 332.8 cm^3^. The planning objectives were on average met for H in the N+ cohort and modestly violated (for V_30Gy_) for HT. The objectives were, on average, not met for the N- patients. The considerable uncertainty (at one standard deviation) is due to the large inter-patient variability in the contouring and in the size of the bowel bags; of course, the interpatient variability in contouring arises from the difference in tumour volumes and ITV generation that represents the real-life situation. Concerning N+ patients, the V_40Gy_ objective was respected in 7/10 cased for H and in 4/10 for HT. V_30Gy_ in 10/10 cases for H and in 7/10 for HT. Concerning N- patients, V_40Gy_ was achieved in 2 patients while V_30Gy_ in 6 patients for the H plans. In the HT case, no patient respected the V_40Gy_ objective and only four the V_30Gy_. As an additional metric, not explicitly enforced in the optimisation process, V_15Gy_ resulted significantly better for HT plans in both N+ and N- subsets.

Concerning the Rectum, on average the soft constraints were met for both H and HT in the N+ set and not met by HT for the N- cases. Halcyon plans allowed some further (and statistically significant for the N- cases) sparing of the rectum compared to the Helical tomotherapy plans. The considerable uncertainty associated with the mean values is primarily due to the inter-patient variability in the position of the rectum with respect to the target volumes. A similar trend, although very modest and not significant, was also observed for the bladder.

The Halcyon plans resulted more conformal than the HT as demonstrated by both the conformality indices with an average gain in the index ranging from 0.1 to 0.4. The CI_Paddick_ for the PTV45 resulted better for HT in the N+ cases (and this was also for the Paddick conformity index computed on the nodal volumes) and slightly better for H in the N- cases.

The estimated treatment time for the HT plans was 3.6 ± 0.3 min and 5.2 ± 0.5 min for the N- and N+ cases respectively. The estimated treatment time for the H plans was 2.4 ± 0.3 and 2.9 ± 0.2 respectively (inclusive of collimator rotation and multiple isocenter setting for the two N+ cases requiring it) since with conventional fractionation the delivery time is mainly given by the rotational velocity of the system which is typically 2 rotations per minute (possibly increasing to 3).

## Discussion

The main scope of this treatment planning investigation was to verify the hypothesis that the new delivery platform Halcyon was adequate to achieve plan quality consistent (equivalent or better) to other consolidated advanced practices and to fulfil stringent clinical protocols. The unique features of Halcyon (mainly the double layer MLC) justify the need for pre-clinical dosimetric investigations to define the field of applicability or the deficiencies of the platform. As a comparison technique, as a novelty factor compared to [13], the Helical Tomotherapy was chosen. The role of HT for cervix uteri was appraised in several studies showing how complex dose distributions with a high degree of conformal avoidance (simultaneous achievement of target coverage and healthy tissue sparing) could be achieved [7–9].

The data summarised in this report confirmed the hypothesis. Halcyon based plans resulted substantially equivalent to Helical Tomotherapy in terms of target volume coverage and allowed for an equivalent or improved sparing of the various OARs. In particular, the sparing of the bowels resulted in about 15–20% further saving (on average ranging from 30 to 60 cm^3^ for V_40Gy_ and V_30Gy_. HT presented, on the contrary, a lower spillage, e.g. measured by V_15Gy_, a predictor of diahrrea related toxicity. This fact allowed to better meet the challenging clinical aims for the bowel (particularly for the N- subset). The quantification of the associated reduction of toxicity should follow proper clinical validation, although it might be hardly detectable with such a modest dose difference. From a speculative point of view, since both parameters are predictors of toxicity, Halcyon plans should be considered advantageous. All other differences in the organs at risk resulted either marginally or not statistically significant, proving the equivalence of H and HT for the remaining metrics.

Kim [19] reported about the treatment of 12 patients treated with extended-field intensity-modulated radiotherapy planned with the dual isocenter technique applied also in the present study for long targets. The dose prescription was equivalent to the Embrace regimen for the N+ patients while the dose-volume objectives for the OARs were different but similarly demanding. Authors demonstrated the capability of Halcyon to generate and deliver the needed high-quality plans for all cases using the dual isocenter approach. The pretreatment dosimetric verification of the plans with a 2D array of ion chambers resulted ranging from 97.3 to 99.9% (with 3%/3 mm thresholds in the distance to agreement and dose difference parameters). The total treatment time was reported in the range of 5 to 6.5 min per fraction.

The potential of Halcyon for cervix uteri was investigated also by Li [20], over a group of 30 patients with a dose prescription of 50.4Gy and no SIB and with dose-volume aims different from the highly demanding Embrace constraints, i.e. in a substantially different setting from what applied in the present investigation. For IMRT plans, authors demonstrated equivalence between Halcyon and c-arm linac based plans for the target volume while achieved improved sparing of organs at risk with H. More importantly, Li reported about the pre-treatment dosimetric verifications of plans. The verification of H plans resulted in an average gamma index passing rate of 99.41 ± 0.26 using 3 mm/3% thresholds in the distance to agreement and dose difference parameters.

De Roover [31] performed validation of intensity-modulated and VMAT plans according to the American Association of Physicists in Medicine task group 119 [32] on some test cases, on anthropomorphic phantoms and on the first patients treated in their clinic. Authors reported gamma agreement scores ranging from 97.0 to 100.0% for the test cases (film dosimetry) and above 95.8% for the patients (diode dosimetry).

The pretreatment verification was out of the scope of our current investigation since HT is a consolidated platform while for H, we assume the validity of the literature data, specific for cervix uteri, reviewed above.

Compared to these two recent investigations, our study focused on the comparative assessment of the potential of Halcyon versus a consolidated technique from the Tomotherapy platform in the frame of the planning rules set by a demanding clinical protocol. All three independent investigations confirm the capability of H to perform at a high level for this challenging clinical indication.

By design, the present study was conducted in double-blind; the results presented are therefore not biased toward one technique. The fundamental impact of the planner’s skills on each technique was mitigated by selecting highly experienced operators for the optimisation of each set of plans.

It is therefore essential to outline the fact that the equivalence/modest superiority of Halcyon was achieved strictly adopting the same clinical dose-volume aims as in an international protocol converted into optimisation constraints tailored to the optimisation engines applied for Halcyon or Helical Tomotherapy.

One of the potential limitations of the Halcyon system was identified in the field length with a single isocenter (28 cm). This study demonstrated that, also in the case of node-positive cervix patients, about 80% of the cases could be treated with one isocenter. The remaining cases were optimised with the automated double isocenter feature of the H system, which allows managing targets as long as 36 cm as positively demonstrated also by Kim [15]. Plans for more extended targets could be optimised with more manual procedures (requiring the use of sequential optimisations). Another potential limitation in the study derives from the fact that dose calculations were performed with 2.5 mm resolution for Halcyon and 5 mm in the x-y plane for Helical Tomotherapy. Although this might be a potential source of inconsistency in the comparison of the two datasets, we appraised its relevance on a subset of 5 patients recalculating the dose distributions in Halcyon with a 5 mm resolution. It resulted that the median percentage difference, computed over all the metrics used in the study, was 0.5% (the mean was 0.7%) ranging from 0.0 to 4.1%. The reason for this derives from the large volumes involved in the study for both targets and OARs, the fact that the voxel size in the HT calculations was < 0.1cm^3^ and that no point metrics were used (consistently with ICRU recommendations [33]). In summary, we concluded that the spatial resolution might bias the results in principle but in a quite modest manner for the presently discussed data. Another limitation factor in the study is the choice of 5 cm as the field aperture which might be considered as a clinically unusual parameter setting (e.g. Hsieh [8] suggests the use of 2.5 cm). The field size can affect the dose conformity and the OAR sparing, particularly in the regions where the PTVs shape changes rapidly in the longitudinal direction. The choice of 5 cm was according to standard clinical practice at the home institute. The most recent tomotherapy units are equipped with the option of dynamic jaw setting. In the present study, fixed jaw was used since clinically available at the institute. The potential (negative) impact of this choice is linked to a broader penumbra region in the cranio-caudal field edges. The use of dynamic jaw would therefore be advisable and might further improve the dose conformity and reduce the dose to some organs at risk (e.g. the bowels).

## Conclusion

This retrospective treatment planning study, based on the dose constraints derived form the Embrace II study protocol, suggested the fundamental equivalence between Halcyon based and Helical Tomotherapy based plans for the intensity-modulated rotational treatment of cervix uteri cancer. Different levels of sparing were observed for the bowels with H better protecting in the high-dose region and HT in the mid-low dose regions. The clinical impact of these differences should be further addressed.

## Data Availability

The datasets used and analysed during the current study are available from the corresponding author.

## Abbreviations

CTV: Clinical target volume
CTV_E: Elective CTV
CTV_HR: High-risk CTV
CTV_LR: Low-risk CTV
CTV_N: Nodal CTV
EBRT: External beam radiation therapy
H: Halcyon
HT: Helical Tomotherapy
IG_IMRT: Image-guided Intensity Modulated Radiation Therapy
IMRT: Intensity Modulated Radiation Therapy
MLC: Multileaf collimator
PTV: Planning target volume
VMAT: Volumetric modulated arc therapy
N+: Node-positive.
N-: Node-negative
CT: Computed tomography
MRI: Magnetic resonance images
GTV: Gross tumour volume
ITV: Internal target volume
ITV45: ITV for the primary tumour
PTV45: PTV for the primary tumour
PTV_N: PTV for the nodal volumes
SIB: Simultaneous integrated boost
OAR: Organ at risk
FFF: Flattening filter free
MU: Monitor units
SSD: Source surface distance
AAA: Anisotropic Analytical Algorithm
DVH: Dose-volume histograms
CI: Conformity index
